# Catheter cryoablation of ventricular ectopy originating from his region

**DOI:** 10.1002/ccr3.2649

**Published:** 2020-02-05

**Authors:** Andrea Rossi, Antonio Frontera, Luca Panchetti, Umberto Startari, Gianluca Mirizzi, Bruno Antonio Formichi, Marcello Piacenti

**Affiliations:** ^1^ The Arrhythmology Unit Department of Invasive Cardiology Fondazione “Gabriele Monasterio” CNR‐Regione Toscana Pisa Italy; ^2^ The Electrophysiology Department Hopital Haut Leveque Bordeaux France

**Keywords:** cryoablation, premature ventricular complexes

## Abstract

Careful mapping, early detection of AV conduction damage and cryothermal energy availability are essential in dealing with ablation procedures at the parahisian region.

## Graphical abstract

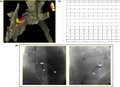

## 1 INTRODUCTION

Catheter ablation of premature ventricular complexes (PVCs) should be considered in patients refractory to pharmacological therapy with disabling symptoms and/or tachycardiomyopathy is induced.^1^, ^2^ Rarely PVCs could originate in proximity of His bundle. In this occurrence, radiofrequency ablation could lead to conduction damage.^3^

## 2 CASE DESCRIPTION

**Figure 1:**
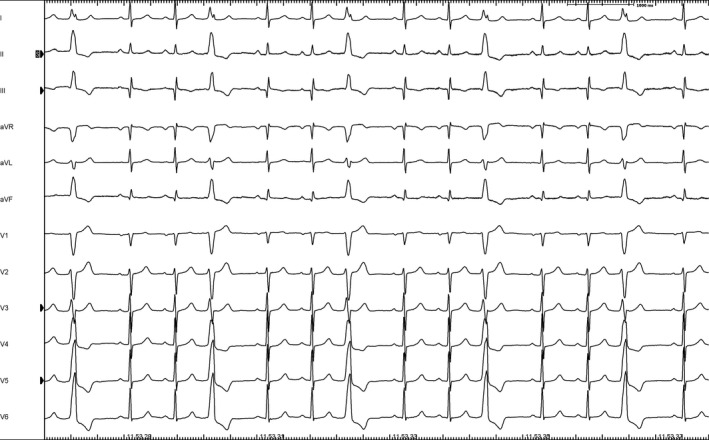
PVCs at the 12 Leads ECG. These were organized in trigeminism

We report the clinical case of a 47‐year‐old man heavily symptomatic for frequent PVCs (40 230 at the holter 24 hours ECG representing 41% of the total beats), refractory to the pharmacological therapy. PVCs showed precordial transition in V3, with monophasic R wave in V4 (Figure 1). Patient was admitted for catheter ablation.

Electrophysiological study with programmed ventricular stimulation did not induce sustained ventricular tachycardias at baseline or during isoproterenol infusion.

**Figure 2:**
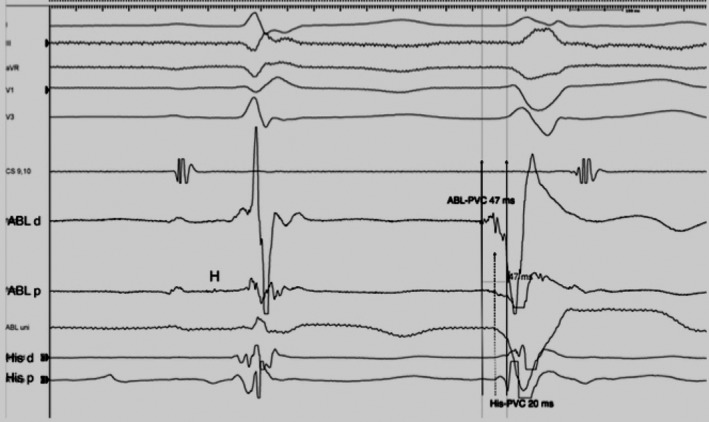
Intracardiac recordings at the site of ablation. Earliest activation was recorded on the ablation catheter placed on the HIS region (47 ms). (ABL = Cryocatheter ablation). See Figure 3, bottom panel for x‐ray location of the catheters

**Figure 3:**
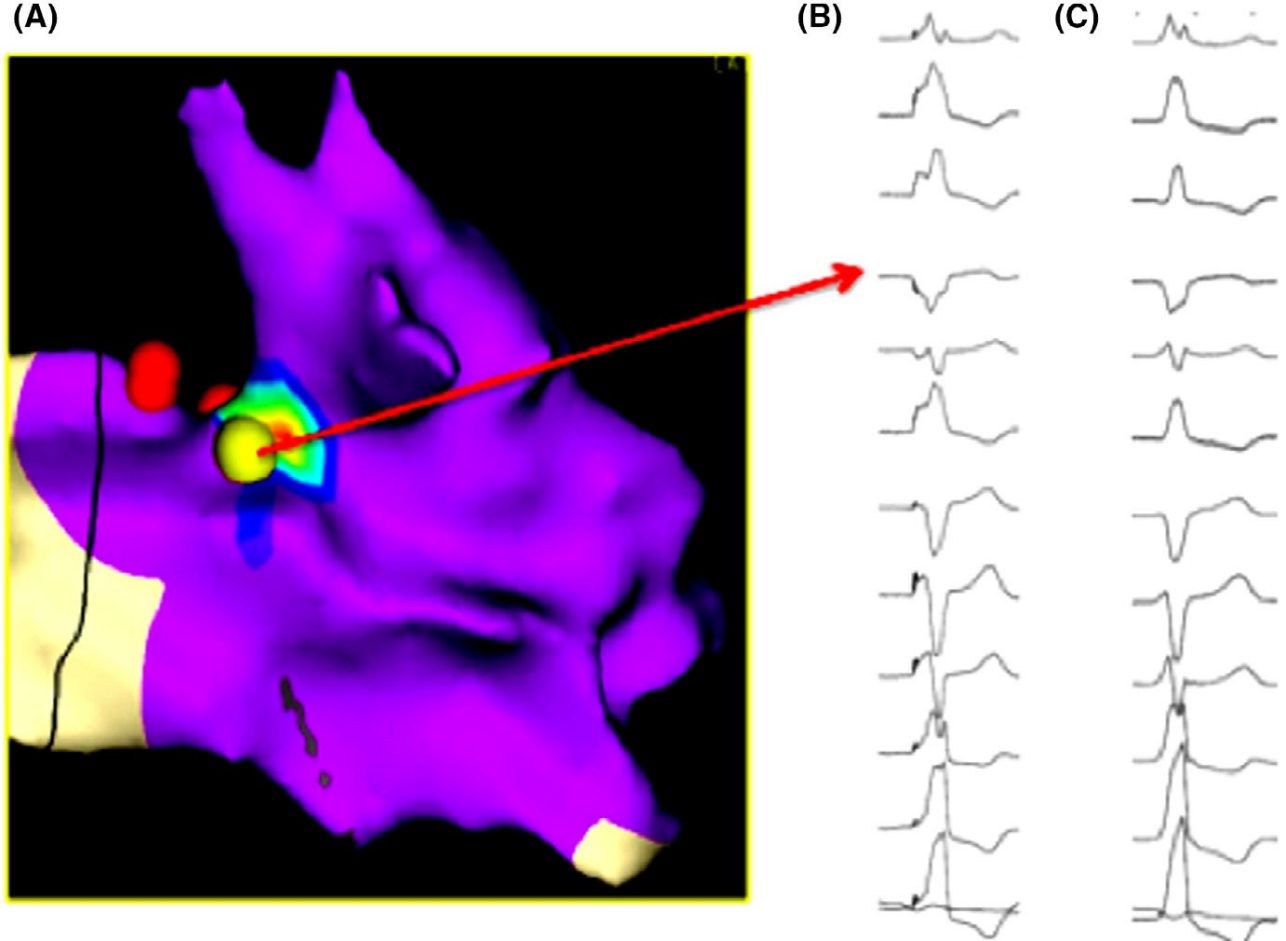
Left panel (A) shows activation map indicating pacemap from successful ablation region (yellow dot); His region on the red dots. Right panel shows perfect match between ventricular pacing from ablation region (B) and clinical ectopic beat (C)

Right ventricular activation mapping was performed with an electroanatomic mapping system (NAVX, St.Jude Medical). The region of earliest activation was recognized at the HIS region, close to the right bundle branch origin, with unipolar QS pattern and timing of −47 ms (Figure 2). Pacemap demonstrated near‐perfect concordance with clinical PVC with 100% concordance (12/12 leads) (Figure 3).

**Figure 4:**
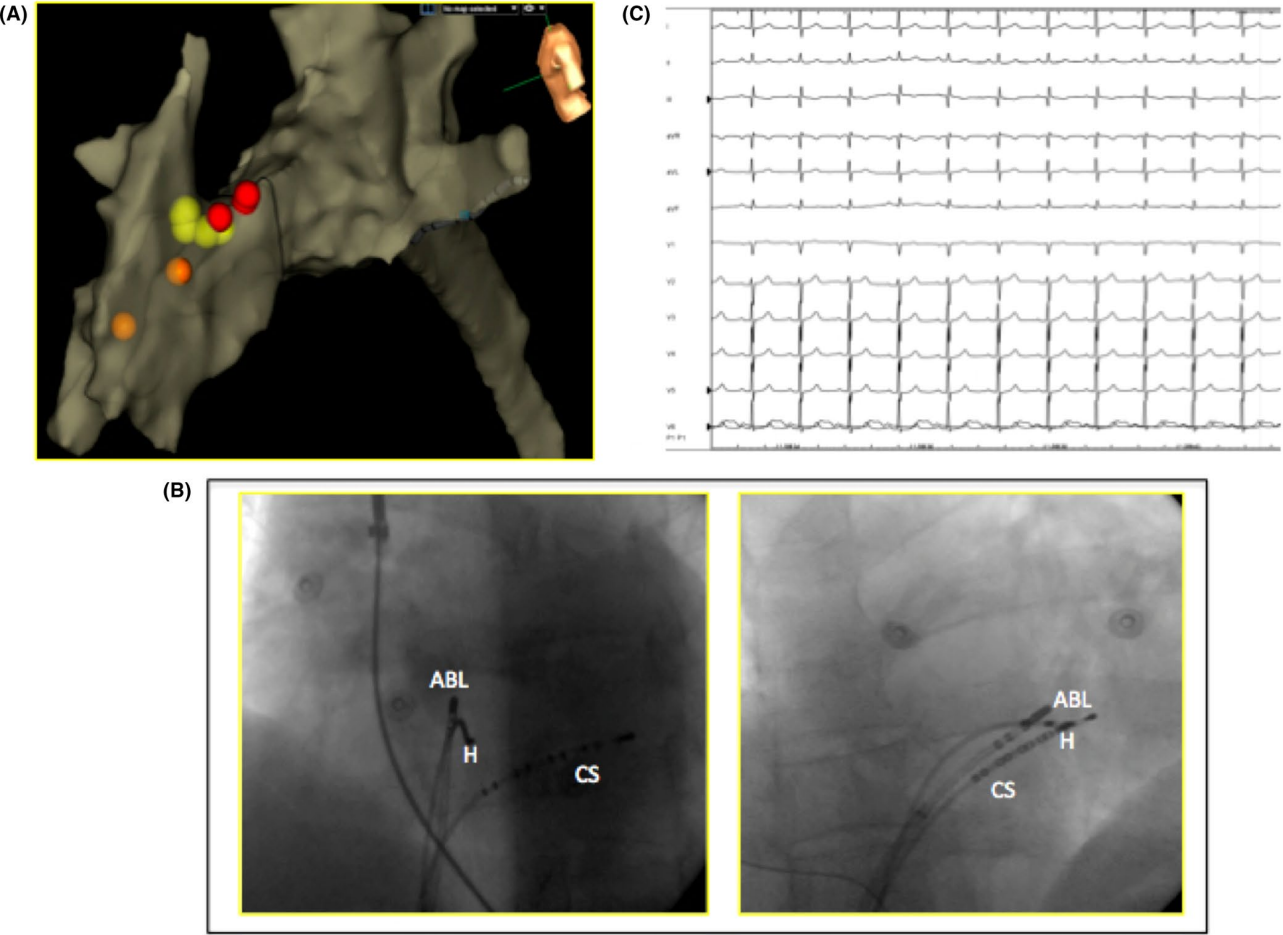
A, Anatomic map of right ventricle with depiction of tricuspidal valve and right atrium. (left latero‐lateral view). Red tags show His location. Orange tags show right bundle branch potentials while yellow dots represent where cryoenergy has been delivered. B, Angiographic LAO and RAO views. C, the electrocardiographic final result after successful cryoablation

Radiofrequency delivery was attempted at this site (20 W 45°C) with abolition of ectopic beats but with temporary right bundle block. In order to avoid persistent conduction damage, a 7‐Fr cryoablator catheter (Metronic) was preferred (6 minutes at −80°C). During this time, AH and HV prolongation and right bundle block occurrence were not appeared (Figure 4). The patient was discharged without complications 2 days after the procedure. Holter ECG 24 hours showed PVCs burden reduced at 4% and 2% at 2 months and 1 year of follow‐up, respectively.

## 3 DISCUSSION

We reported a successful catheter cryoablation of PVCs originating from the Hisian region without AV conduction damage. When monomorphic, repetitive, premature ventricular beats have the characteristics of vertical axis with notched R wave in D1, rSr’ on AVL and QS pattern in AVR, precordial transition was in V3, a focus close to the Hisian region should be suspected. Given the fragility of this region and the effects of RF energy delivery on the right bundle, cryothermal energy should be always contemplated leading to an effective ectopy suppression without right bundle block creation due to a very superficial lesion not leading to right bundle branch block.

Safety and feasibility of parahisian cryoablation for ventricular arrhythmias is well reported in the literature. Several papers described cases of successful ablation procedures using irrigated and nonirrigated RF energy.^4^, ^5^ Di Biase et al reported the largest population of 7 cases of ventricular arrhythmias originating from His region treated with successful cryoablation. No complication or failure was mentioned.

This case report highlights the importance to recognize the correct approach during the ablation setting in such difficult situations. Careful mapping, early detection of AV conduction damage, and cryothermal energy availability are essential in dealing with ablation procedures at the parahisian region.

## CONFLICT OF INTEREST

None.

## AUTHOR CONTRIBUTIONS

AR: From the Arrhythmology Unit, Department of Invasive Cardiology, Fondazione “Gabriele Monasterio” CNR‐Regione Toscana, Pisa, Italy. Corresponding Author. Electrophysiology Staff. AF: From the Electrophysiology Department, 1 avenue Magellan, Hopital Haut Leveque, Bordeaux, France. Co‐Author. Precious role in Case‐Report writing. LP: From the Arrhythmology Unit, Department of Invasive Cardiology, Fondazione “Gabriele Monasterio” CNR‐Regione Toscana, Pisa, Italy. Co‐Author. Electrophysiology Staff. US: From the Arrhythmology Unit, Department of Invasive Cardiology, Fondazione “Gabriele Monasterio” CNR‐Regione Toscana, Pisa, Italy. Co‐Author. Electrophysiology Staff. GM: From the Arrhythmology Unit, Department of Invasive Cardiology, Fondazione “Gabriele Monasterio” CNR‐Regione Toscana, Pisa, Italy. Co‐Author. Electrophysiology Staff. BAF: From the Arrhythmology Unit, Department of Invasive Cardiology, Fondazione “Gabriele Monasterio” CNR‐Regione Toscana, Pisa, Italy. Co‐Author. Anesthesiologist. MP: From the Arrhythmology Unit, Department of Invasive Cardiology, Fondazione “Gabriele Monasterio” CNR‐Regione Toscana, Pisa, Italy. Co‐Author. Chief of Electrophysiology Department.
